# SecretSanta: flexible pipelines for functional secretome prediction

**DOI:** 10.1093/bioinformatics/bty088

**Published:** 2018-02-16

**Authors:** Anna Gogleva, Hajk-Georg Drost, Sebastian Schornack

**Affiliations:** Sainsbury Laboratory, University of Cambridge, Cambridge, UK

## Abstract

**Motivation:**

The secretome denotes the collection of secreted proteins exported outside of the cell. The functional roles of secreted proteins include the maintenance and remodelling of the extracellular matrix as well as signalling between host and non-host cells. These features make secretomes rich reservoirs of biomarkers for disease classification and host–pathogen interaction studies. Common biomarkers are extracellular proteins secreted via classical pathways that can be predicted from sequence by annotating the presence or absence of N-terminal signal peptides. Several heterogeneous command line tools and web-interfaces exist to identify individual motifs, signal sequences and domains that are either characteristic or strictly excluded from secreted proteins. However, a single flexible secretome-prediction workflow that combines all analytic steps is still missing.

**Results:**

To bridge this gap the *SecretSanta* package implements wrapper and parser functions around established command line tools for the integrative prediction of extracellular proteins that are secreted via classical pathways. The modularity of *SecretSanta* enables users to create tailored pipelines and apply them across the whole tree of life to facilitate comparison of secretomes across multiple species or under various conditions.

**Availability and implementation:**

*SecretSanta* is implemented in the R programming language and is released under GPL-3 license. All functions have been optimized and parallelized to allow large-scale processing of sequences. The open-source code, installation instructions and vignette with use case scenarios can be downloaded from https://github.com/gogleva/SecretSanta.

**Supplementary information:**

Supplementary data are available at *Bioinformatics* online.

## 1 Introduction

Secreted proteins play a vital role in interactions between neighbouring cells within the same organism as well as between different species engaged in close contact. Their role is pivotal in the context of host–pathogen interactions, where numerous virulence factors are secreted to facilitate recognition and colonization of host cells and tissues. Hence, robust prediction of secreted proteins is usually the first step towards the informed development of efficient infection control strategies.

Secretome prediction requires multiple steps. In the first step, short signal peptides are determined at the N-terminal end of a protein sequence. Next, it is crucial to ensure the absence of specific sequence motifs and domains that target the protein to specific organelles or prevent it from being secreted. Such sequences include transmembrane domains, endoplasmic reticulum (ER) lumen retention signals and mitochondria/plastid targeting signals.

A variety of command line tools and web-interfaces are available to perform predictions of individual motifs and domains, such as SignalP (Nielsen *et al*., 1997) for the prediction of signal peptides; TargetP (Emanuelsson *et al*., 2000) and WolfPsort (Horton *et al*., 2006) for the prediction of subcellular localization; TMHMM (Sonnhammer *et al*., 1998) and TOPCONS (Tsirigos *et al*., 2015)—for the prediction of transmembrane domains. However, an interface that combines these tools in a single flexible workflow for standardized and efficient secretome prediction was previously unavailable.

*SecretSanta* bridges this gap by providing a set of optimized wrapper functions around a variety of command line tools. These functions are designed to generate tidy data output to seamlessly integrate into the R data science infrastructure. Users can pipe intermediate outputs between individual methods, run resource-demanding stages in parallel, compare predictions between similar methods and trace back selected steps of the pipeline. Taken together, *SecretSanta* provides a platform to build reproducible and flexible pipelines for scalable secretome prediction. This framework can facilitate comparisons of secretomes across multiple species or under various conditions and can potentially lead to the discovery of novel classes of previously neglected secreted proteins.

## 2 Implementation

*SecretSanta* is released under GPL-3 license. The source code along with detailed usage guidelines and unit tests is publicly available at https://github.com/gogleva/SecretSanta. *SecretSanta* requires a number of external dependencies: *SignalP 2.0*, *SignalP 3.0*, *SignalP 4.1*, *TargetP 1.1*, *TMHMM 2.0c*, *WoLF PSORT* which are freely available for academic users. It also depends on several R packages: *Biostrings*, *dplyr*, *parallel*, *reardr*, *tibble*, *stringr* and *biomartr* package (Drost and Paszkowski, 2017) for automated retrieval of proteomes. The *SecretSanta* package contains wrapper functions around the external dependencies as well as the check_khdel() function to scan for C-terminal ER-retention signals, the m_slicer() function to generate peptides with alternative translation start sites and the convenient ask_uniprot() function to retrieve known information on protein subcellular localization from UniprotKB. All *SecretSanta* functions are designed to work together by producing standardized output as an instance of the superclass CBSResult. Objects of CBSResult class contain an in_fasta slot storing the submitted sequences and an out_fasta slot storing positive candidates after the prediction run. Each particular method then complements CBSResult simple structure with relevant slots. For example, outputs of the signalp() function are organized in SignalpResult objects. In addition to inherited in_fasta and out_fasta slots, SignalpResult objects contain three additional slots, relevant for the prediction of signal peptides:

- sp_tibble—parsed SignalP tabular output for positive candidates;
- mature_fasta—sequences with cleaved signal peptides;
- sp_version—version of SignalP software used.

The underlying class structure allows the user to easily pipe results between individual methods to create flexible custom pipelines and also to compare results generated with similar methods. Piping could be done either explicitly, by specifying output object from the previous analytic step as an input to the next, or—implicitly—by using the pipe operator %>% provided by the *magrittr* package. Finally, processing of large input *fasta* files can be run in parallel making them suitable for HPC usage.

## 3 Usage

To illustrate the core functionality of the SecretSanta package we will establish a minimal pipeline with the following analytic steps:

- use SignalP-4.1 to predict signal peptides and cleavage sites;
- integrate TOPCONS predictions to ensure that proteins do not contain transmembrane domains; note, the tmhmm() function could also be used, we recommend TOPCONS for more accurate results;
- run TargetP on the output to make sure that the set of selected proteins is not targeted to plastids or mitochondria;
- collect the TargetP output and scan for ER-retention signals.

First we read a short sample *fasta* file and initialize an instance of CBSResult class:

input <- CBSResult ( in_fasta =  rea dAAStringSet (‘proteins.fasta’ ))

Next, we start our pipeline by running the signalp() function:

a <- signalp(input, version = 4, organism = ’euk’, run_mode = ’starter’)

Now we can use the a object as an input to the topcons() parser to integrate prediction of transmembrane domains.

b <- topcons (a, TM = 0, p_dir = ’rst.zip’, topcons_mode = ’WEB-server’)

To check for potential mitochondria or plastid targeting signals, we will apply targetp() function to the resulting b object. Please note, that targetp() will use out_fasta slot with the full sequence as an input:

c <- targetp(b, network = ’N’, run_mode = ’piper’)

Finally, we will make sure that there are no ER-retention signals, defined according to the PROSITE model:

d <- check_khdel(c, pattern = ’prosite’)

More detailed tutorials are available in the online Supplementary Material.

## 4 Conclusion

*SecretSanta* functions provide an integrated new way to predict and analyze secretomes. The flexibility and modularity of the pipelines grants analytical freedom and computational reproducibility to fuel comparative studies of secreted proteins across the tree of life.

## Supplementary Material

Supplementary Data
