# Supplementary material for: SecretSanta: flexible pipelines for functional secretome prediction
Source: Bioinformatics. 2018 Feb 16;34(13):2295–6. doi: 10.1093/bioinformatics/bty088 (PMC6022548; doi:10.1093/bioinformatics/bty088)
Supplement: Supplementary Data [file bty088_secretsanta_vignette_revised_12_02_2018.pdf]

SecretSanta: flexible pipelines for functional secretome prediction

Anna Gogleva

2018-02-12 16:47:32

1. Background

The **SecretSanta** package provides an R interface for the integrative prediction of extracellular proteins that are secreted via classical pathways.

Secretome prediction often involves multiple steps. Typically, it starts with identification of short signal peptides at the N-terminal end of a protein (**Figure 1 a**). Next, it is crucial to ensure the absence of motifs and domains preventing the protein from being secreted or targeting it to specific organelles. Such sequences include transmembrane domains, short ER lumen retention signals and mitochondria/plastid targeting signals (**Figure 1 b-d**). The ultimate aim of a secretome prediction pipeline is to identify secreted proteins as shown in **Figure 1 a** and filter out those shown in **Figure 1 b-d**.

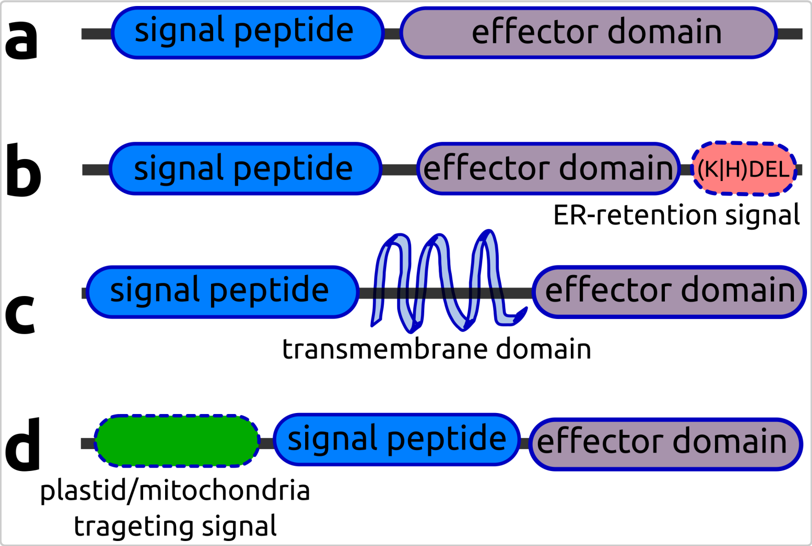

Figure 1. Characteristic motifs, domains and their arrangements, distinguishing extracellular proteins from proteins retained inside the cell.

A number of command line tools and web-interfaces are available to perform predictions of individual motifs and domains (SignalP, TargetP, TMHMM, TOPCONS, WoLF PSORT), however the interface allowing to combine the outputs in a single flexible workflow is lacking.

The **SecretSanta** package attempts to bridge this gap. It provides a set of wrapper and parser functions around existing command line tools for predictions of signal peptides and protein subcellular localization. The functions are designed to work together by producing standardized output as an instance of `CBSResult` class.

Objects of `CBSResult` class contain an `in_fasta` slot with the initial submitted sequences and an `out_fasta` slot with positive candidates after the method application. Within `CBSResult` class objects fasta slots are organised in `AAStringSetList`. `in_fasta` and `out_fasta` slots could be extracted separately with designated accessor functions: `getInfasta` and `getOutfasta`. Alternatively, all fasta? slots could be extracted at once, organised in `AAStringSetList` object, with `getFastas` method. This enables application of all the defined `AAStringSetLisst` methods (e.g., `elementNROWS`) to the extracted object and ensures

seamless integration into numerous workflows, utilising this core Bioconductor class. Example below illustrates basic manipulations with objects of `CBSResult` class.

```
aa <- readAAStringSet(system.file("extdata", "sample_prot_100.fasta", package =
"SecretSanta"))

# create a simple CBSResult object:
inp <- CBSResult(in_fasta = aa,
                 out_fasta = aa[1:10])
class(inp)
```

```
## [1] "CBSResult"
## attr(,"package")
## [1] "SecretSanta"
```

```
inp
```

```
## An object of class CBSResult
##  in_fasta out_fasta
##       100        10
```

```
# access both fasta slots at once:
getFastas(inp)
```

```
## AAStringSetList of length 2
## [["in_fasta"]] ALI_PLTG_1 Hypothetical protein _1_624_ 908_+=MVEPSSPECVV...
## [["out_fasta"]] ALI_PLTG_1 Hypothetical protein _1_624_ 908_+=MVEPSSPECV...
```

```
# access in_fasta slot:
getInfasta(inp)
```

```
##   A AAStringSet instance of length 100
##       width seq                                         names
##   [1]    94 MVEPSSPECVVQIIKDEKLK...LLVCGFIYFAVLQSWLLES ALI_PLTG_1 Hypoth...
##   [2]   240 MTRQGYLIVHDKRSRPTVRY...VTPHYVPTHFGRGNNQEAH ALI_PLTG_2 Unknow...
##   [3]   533 MQPTSSSAPQSDVVVADPPS...QQQQEQQANSTALKNERSI ALI_PLTG_3 Hypoth...
##   [4]   456 QFLSELRKVSVSTKLDGVQR...QSQQNSRPREGCCSSCIIF ALI_PLTG_4 Hypoth...
##   [5]   210 MKLSSVCTIIFGLVFIDFNN...DGSVTTAAQKAAALVKANA ALI_PLTG_5 Cutin ...
##   ...   ... ...
##  [96]   650 MQRLVQRDKDAPVVREGDDD...DRLSLNALLEAFPPMNSRL ALI_PLTG_96 Prote...
##  [97]   236 MSTKRSRSAPILRVKKLTPE...DDDNSAKVLEWINAFIESN ALI_PLTG_97 Deoxy...
##  [98]   239 MSLNYLAHTNMRRARENFLK...KTYIEYEAVENNTLNKTEG ALI_PLTG_98 Hypot...
##  [99]   566 MCTSFSDQEDKKLVVLATEY...PRLDRKFFGLQVAPPPTSQ ALI_PLTG_99 Histo...
## [100]   180 MDNYYTSVQLLLDLQLKGLC...DPHRTFLTDLVGVELQVMG ALI_PLTG_100 Tran...
```

```
# access out_fasta slot:
getOutfasta(inp)
```

```
##   A AAStringSet instance of length 10
##       width seq                                               names
##  [1]    94 MVEPSSPECVVQIIKDEKLK...TLLVCGFIYFAVLQSWLLES ALI_PLTG_1 Hypoth...
##  [2]   240 MTRQGYLIVHDKRSRPTVRY...AVTPHYVPTHFGRGNNQEAH ALI_PLTG_2 Unknow...
##  [3]   533 MQPTSSSAPQSDVVVADPPS...QQQQQEQQANSTALKNERSI ALI_PLTG_3 Hypoth...
##  [4]   456 QFLSELRKVSVSTKLDGVQR...NQSQQNSRPREGCCSSCIIF ALI_PLTG_4 Hypoth...
##  [5]   210 MKLSSVCTIIFGLVFIDFNN...SDGSVTTAAQKAAALVKANA ALI_PLTG_5 Cutin ...
##  [6]   177 MPGLGICGEPLVSGITSNLG...TAAQKAAALVQGGSARALRG ALI_PLTG_6 Cutin ...
##  [7]   176 MAGLGICGEPLVSGITSDLS...TTAAQKAAALVKNSARILRA ALI_PLTG_7 Cutin ...
##  [8]   155 MSVSSYAVSYLASMDQTSAG...TTAAQKAAALVQGGARKLRM ALI_PLTG_8 Cutin ...
##  [9]  2095 MAGLGICGEPLVSGITSDLS...IAAKNQEEPKERERPECAQQ ALI_PLTG_9 Kinesi...
## [10]   260 MQHDEVIWSVISKQFCSFKS...PYVEIEYEQEQETATGELNW ALI_PLTG_10 Putat...
```

Each particular method then complements this simple class structure with relevant slots. For example, outputs of the `signalp()` function are organised in `SignalpResult` objects. Apart from `in_fasta` and `out_fasta` slots inherited from `CBSResult` class, `SignalpResult` objects contain three additional slots, relevant for the **SignalP** method:

- `sp_tibble` - parsed **SignalP** tabular output for positive candidates;
- `mature_fasta` - mature sequence for the candidate secreted proteins, i.e sequences with cleaved N-terminal signal peptides;
- `sp_version` - version of **SignalP** used to generate this object.

The detailed organisation of class structure is shown in the **Figure 2**.

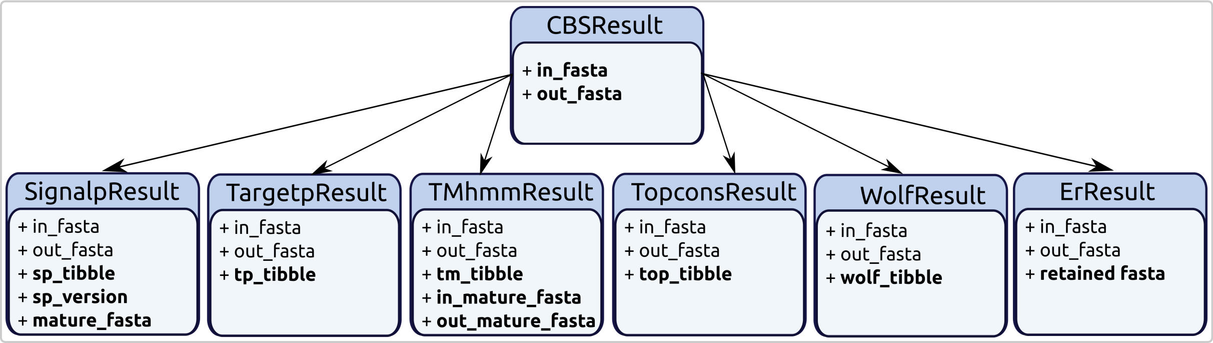

Figure2. SecretSanta S4 class structure.

This uniform class organisation allows the user to easily pipe results between individual predictors to create flexible custom pipelines and also to compare predictions between similar methods. For instance, between **TargetP** and **WoLF PSORT** for subcellular localization and between multiple versions of **SignalP** for signal peptide prediction.

To speed-up processing of large input fasta files initial steps of the pipeline are automatically run in parallel when the number of input sequences exceeds a certain limit. The package also contains a convenient `ask_uniprot()` function - to export known information about subcellular localisation. This allows the user to compare and verify predictions of extracellular proteins, however is mostly applicable for well-annotated and curated genomes.

Taken together **SecretSanta** provides a platform to build automated multi-step secretome prediction pipelines that can be applied to large protein sets to facilitate comparisons of secretomes across multiple species or under various conditions. For example, an earlier draft of this package was successfully used to compare the secreted protein complement of a plant pathogen throughout an infection time-course in plant roots (Evangelisti *et al.*, 2017).

To avoid confusion, the names of external command line tools will be shown in **bold**, and the corresponding R functions from the **SecretSanta** package will have standard `code highlighting`. For example:

- **SignalP** - CBS SignalP and its associated derivative files;

- `signalp()` - **SecretSanta** wrapper function around **SignalP**;

Majority of the **SecretSanta** functions could be run in 2 modes:

- `starter` - when initiating the secretome pipeline with the above function;
- `piper` - for downstream/intermediate steps, so that the function expects an output from another wrapper function. For detailed information on piper/starter behavior please see Table 2.

2. Installation of external dependencies

SecretSanta relies on a set of existing command line tools to predict secreted proteins. Please install them and configure according to the listed instructions. Due to limitations imposed by the external dependencies, some of **SecretSanta** wrapper functions are fully functional only on Linux systems.

2.1 Download and configure external dependencies

Tools for prediction of signal peptides and cleavage sites:

- **SignalP 2.0**
  - This version can run under IRIX, IRIX64, Linux, OSF1, SunOS.
  - Download stand alone **SignalP 2.0** http://www.cbs.dtu.dk/cgi-bin/sw_request?signalp+2.0
  - Unpack the archive

```
tar -zxvf signalp-2.0.Linux.tar.Z
cd signalp-2.0
```

  - Edit “General settings” at the top of the **signalp** file. Set value of ‘SIGNALP’ variable to be path to your **signalp-2.0** directory. Other variables usually do not require changes. We will not use plotting functions from *SignalP*, so gnuplot, ppmtogif and ghostview are not required. For more details please check `signalp-2.0.readme`.
  - Since we want to be able to run different versions of **SignalP**, including the legacy versions, it is important to be able to discriminate between them. R is oblivious to shell aliases, so we will simply rename the **signalp** script:

```
mv signalp signalp2
```

- **Signalp 3.0**
  - This version will run on the most common UNIX platforms.
  - Download stand alone **Signalp 3.0** http://www.cbs.dtu.dk/cgi-bin/sw_request?signalp+3.0
  - Unpack the archive

```
tar -zxvf signalp-3.0.Linux.tar.Z
cd signalp-3.0
```

  - Similar to **Signalp 2.0**, edit “General settings” at the top of the **signalp** file. Set value of ‘SIGNALP’ variable to be path to your **signalp-3.0** directory. Other variables usually do not require changes. For more details please check `signalp-3.0.readme`.
  - Rename **signalp** script to avoid further confusion between the versions:

```
mv signalp signalp3
```

- **SignalP 4.1** - the most recent version
  - This version can run under Windows, OS X (Macintosh) and Linux.
  - Download stand alone **SignalP 4.1** http://www.cbs.dtu.dk/cgi-bin/nph-sw_request?signalp

- Unpack the archive

```
tar -zxvf signalp-4.1.Linux.tar.Z
cd signalp-4.1
```

- Edit “General settings” at the top of the **signalp** file. Set values for ‘SIGNALP’ and ‘outputDir’ variables. For more details please check `signalp-4.1.readme`.
- Rename **signalp** script to avoid further confusion between the versions:

```
mv signalp signalp4
```

Tools for prediction of protein subcellular localization:

- **TargetP 1.1**
  - **TargetP 1.1** will run on the most common UNIX platforms
  - Download stand alone **TargetP 1.1**: http://www.cbs.dtu.dk/cgi-bin/nph-sw_request?targetp
  - Unpack the archive:

```
tar -zxvf targetp-1.1b.Linux.tar.Z
cd targetp-1.1
```

  - Edit the paragraph labeled “GENERAL SETTINGS, customize” at the top of the **targetp** file. Set values for ‘TARGETP’ and ‘TMP’ variables. Ensure, that the path to **TargetP** does not exceed 60 characters, otherwise **TargetP 1.1** might fail.
- **WoLF PSORT**
  - Clone **WoLF PSORT**

```
git clone https://github.com/fmaguire/WoLFPSort.git
cd WoLFPSort
```

  - Copy the binaries from the appropriate platform specific binary directory `./bin/binByPlatform/binary-?` to `./bin/`
  - For more details please check the `INSTALL` file.
  - The most important script we need **runWolfPsortSummary** has a bulky name, we will rename it to **wolfpsort** for future convenience:

```
mv runWolfPsortSummary wolfpsort
```

Tools for predicting transmembrane domains

- **TMHMM 2.0**
  - *TMHMM 2.0** will run on the most common UNIX platforms
  - Download stand alone tmhmm (http://www.cbs.dtu.dk/cgi-bin/nph-sw_request?tmhmm)
  - Unpack the archive:

```
tar -zxvf tmhmm-2.0c.Linux.tar.gz
cd tmhmm-2.0c
```

  - Set correct path for Perl 5.x in the first line of `bin/tmhmm` and `bin/tmhmmformat.pl` scripts.
  - For more details please check the `README` file.

2.2 Organise access to the external dependencies

Two options are possible:

Option A: all the external dependencies are accessible from any location.

If you follow this route, there will be no need to provide paths to the respective tool when running **SecretSanta** functions. For bash users this requires modification of `$PATH` environment variable. To make the change permanent, edit `.profile`:

Open ./profile:

```
gedit ~/.profile
```

Add a line with all the path exports. In this example all the dependencies are installed in the `my_tool` directory:

```
export PATH=
"/home/my_tools/signalp-4.1:\
/home/my_tools/signalp-2.0:\
/home/my_tools/signalp-3.0:\
/home/my_tools/targetp-1.1:\
/home/tmhmm-2.0c/bin:\
/home/my_tools/WoLFPSort/bin:\
$PATH"
```

Reload `.profile`:

```
. ~/.profile
```

Reboot to make changes visible to R.

If you are using csh or tcsh, edit `.login` instead of `.profile` and use the `setenv` command instead of `export`.

To check that all the tools are installed correctly, we will use `manage_paths()` function, it runs small test jobs to verify that all the external dependencies are functional and will not cause downstream problems.

```
library(SecretSanta)
```

To run checks for all the dependencies:

```
check_all <- manage_paths(in_path = TRUE)
```

It is also possible to run just a single test for a specific dependency in question:

```
check_small <- manage_paths(in_path = TRUE, test_tool = 'signalp2')
## Checking dependencies accessible via $PATH ...
## No path has been specified.
## signalp2  run completed!
```

Option B: paths to external dependencies are supplied in a separate file:

This option should be used when `$PATH` variable can not be modified. If you follow this route, please specify path to the respective tool executable when running individual wrappers via `paths` argument.

To supply all the paths at once, create a 2-column space-separated text file without headers with listed full paths for all the external dependencies. Please note, when providing path file you can avoid renaming executable for multiple versions of **SignalP**.

Table1: Sample paths to external dependencies

tool	path
signalp4	/home/tools/signalp-4.1/signalp
signalp3	/home/tools/signalp-3.0/signalp
signalp2	/home/tools/signalp-2.0/signalp
targetp	/home/tools/targetp-1.1/targetp
TMHMM	/home/tools/tmhmm-2.0c/bin/tmhmm
WoLFPSORT	/home/tools/WoLFPSort-master/bin/wolfpsort

For external tool names please use simple character strings without version numbers. The only exception here are different versions of **SignalP**, which require version number in one-digit format in the `tool` column to be distinguishable.

Now, we can check the dependencies using `manage_paths()` function. In this case we need to provide value for the `path_file` argument and set `in_path = FALSE`. Note: `manage_paths()` is case insensitive and will convert all the tool names provided in the file to the lower case.

```
check2 <- manage_paths(
    in_path = FALSE,
    path_file = system.file("extdata",
    "sample_paths",
    package = "SecretSanta")
    )
```

3. Pipers and starters

Here is a short summary of individual methods available via the **SecretSanta** package:

Table2: Summary of individual methods.

tool	function_name	purpouse	organisms	starter	piper	parallelised
Signalp-2.0	signalp	predict signal peptides	eukaryotes, gram+, gram-	Yes	Yes	Yes
Signalp-3.0	signalp	predict signal peptides	eukaryotes, gram+, gram-	Yes	Yes	Yes
Signalp-4.1	signalp	predict signal peptides	eukaryotes, gram+, gram-	Yes	Yes	Yes
Targetp-1.1	targetp	predict subcelluar localisation	plants, not plants	Yes	Yes	Yes
wolfpsort	wolfpsort	predict subcelluar localisation	plant, animal, fungi	Yes	Yes	No
Tmhmm-2.0	tmhmm	predict transmembrane domains	all taxons	No	Yes	No

tool	function_name	purpouse	organisms	starter	piper	parallelised
TOPCONS2	topcons	predict transmembrane domains	all taxons	No	Yes	No
NA	check_khdel	scan for ER-retention motifs	eukaryotes	No	Yes	No
NA	m_slicer	generate sequences with alternative translation start sites	all taxons	Yes	Yes	No

4. Individual methods

4.1 Signalp()

SignalP is a software tool to predict classical signal peptides and cleavage sites (Nielsen *et al.*, 1997) in eukaryotes and bacteria. The method combines prediction of signal peptides and cleavage sites based on a combination of artificial neural networks and hidden Markov models. The latter can distinguish between signal peptides and non-cleaved signal anchors (Nielsen and Krogh, 1998).

Currently `signalp()` function from **SecretSanta** provides an interface for the three recent versions of **SignalP**. By providing access for legacy versions of **SignalP** - 2.0 (Nielsen and Krogh, 1998) and 3.0 (Bendtsen *et al.*, 2004), we allow the user to perform comparisons with already existing secretome datasets, predicted with the same method versions. The most recent **Signalp 4.1** (Petersen *et al.*, 2011) version, applied with default thresholds could be not sensitive enough to predict certain classes of secreted oomycete and fungal effectors (Sperschneider *et al.*, 2015). Similar, to the web-service and command line tool, there is a way to run **signalp** in the sensitive mode when 4.1 version is used, by specifying `sensitive = TRUE`.

`signalp()` requires providing `organism` argument; possible values include:

- *'euk'* - for eukaryotes;
- *'gram+'* - for gram-positive bacteria;
- *'gram-'* - for gram-negative bacteria.

When legacy versions of **SignalP** are used, 2.0 and 3.0, please provide a value for the `legacy_method` argument. There are two possible options, often producing quite different results:

- *'hmm'* - for HMM-based predictions;
- *'nn'* - for predictions based on neural networks.

To run `signalp()` prediction, first read fasta file with amino acid sequences and store its contents in a separate variable:

```
aa <- readAAStringSet(system.file("extdata", "sample_prot_100.fasta",
                                  package = "SecretSanta"))
```

Initialize object of `CBSResult` class with `aa` as `in_fasta` slot.

```
inp <- CBSResult(in_fasta = aa)
inp
```

```
## An object of class CBSResult
##  in_fasta out_fasta
##       100         0
```

Since this is the first step in our secretome prediction pipeline, we will run `signalp()` in a *starter* mode. Here we select version number 4 (**SignalP 4.1**) and assume that **SignalP** is accessible globally, so no path files should be specified. If **SignalP 4.1** can not be accessed globally, please provide a relevant path via `paths` argument. For more details please see **Option B**. Please note that `signalp()` truncates all the input sequences longer than 2000 a.a, by default. In this case '_truncated' is added to the original sequence ids to keep track of the changes. An alternative option is to discard long sequences completely before the analysis, to do so set `truncate = FALSE` when running `signalp()`.

```
step1_sp4 <- signalp(inp, version = 4,
                     organism = 'euk',
                     run_mode = "starter")
## Version used ... signalp4
## Running signalp locally ...
## 2  sequences need to be truncated ...
## Ok for single processing.
## Submitted sequences... 100
## Candidate sequences with signal peptides ... 5
```

The above code under the hood runs **SignalP 4.1** and outputs result as an instance of `SignalpResult` class, which inherits from a parental `CBSResult()`class.

```
class(step1_sp4)
```

```
## [1] "SignalpResult"
## attr(,"package")
## [1] "SecretSanta"
```

The `SignalpResult` object contains 5 slots:

- `in_fasta` - original set of input amino acid sequences;
- `out_fasta` - full length amino acid sequences of positive candidates;
- `mature_fasta` - mature sequence for the candidate secreted proteins, i.e sequences with cleaved N-terminal signal peptides;
- `sp_tibble` - parsed **SignalP** tabular output for positive candidates;
- `sp_version` - version of **SignalP** used to generate this object.

You can use accessor methods to get contents of individual fasta slots:

```
getInfasta(step1_sp4)
```

```
##   A AAStringSet instance of length 100
##       width seq                                         names
##   [1]   239 MSLNYLAHTNMRRARENFLK...KTYIEYEAVENNTLNKTEG ALI_PLTG_98
##   [2]   275 MATNPPTVAPVELRELNFAF...NRAGGYAAGRLGGVDIDSV ALI_PLTG_47
##   [3]   169 EQQHELLTCLGGTLTGTQKN...RQYIAMTGDVKLTDALKQL ALI_PLTG_82
##   [4]   119 MLQATTSKDARQPLHVDTNN...LMQLKRRRSMNLSHPSLFK ALI_PLTG_44
##   [5]   172 MRSYLAKHPRYPKAAPTKAT...HIRTRLAYASTMLKYSNKD ALI_PLTG_91
##   ...   ... ...
##  [96]   168 MRLPEAHHWDAAARSESKSL...IWYLPLFAFLKTMKFCKLI ALI_PLTG_61
##  [97]   207 MYDMKSDVDARVMNYFHLCN...VLIPELKFRVLFYDTKKDW ALI_PLTG_80
##  [98]   842 MAGDFGTKTTTLCARTRLRS...LGCLLYELMTGQVCTLISS ALI_PLTG_45
```

```
##  [99]   336 MKTDIGSTSPSYGSYAVLVP...EDPEREEVVAYVLRWLRLQ ALI_PLTG_21
## [100]   210 MKLSSVCTIIFGLVFIDFNN...DGSVTTAAQKAAALVKANA ALI_PLTG_5
```

```
getOutfasta(step1_sp4)
```

```
##   A AAStringSet instance of length 5
##     width seq                                          names
## [1]   118 MRLKFSILIFGAVLLATTTNA...NIRYASMLQDFLNTYHRRGV ALI_PLTG_72
## [2]   653 MKIVALVTFCIATLDSSIVFA...SEDPVYPLVKEYSDVVSKHP ALI_PLTG_23
## [3]   495 MVVRVLLVVLALLAVGVQSKA...FAIVTIGASDGRVRYMNPPS ALI_PLTG_38
## [4]   164 MVSTSRVFALLLLPSPSARIF...SMVEHIKTTKRVIDEVQDHV ALI_PLTG_83
## [5]   210 MKLSSVCTIIFGLVFIDFNNV...SDGSVTTAAQKAAALVKANA ALI_PLTG_5
```

```
getMatfasta(step1_sp4)
```

```
##   A AAStringSet instance of length 5
##     width seq                                          names
## [1]    97 DDNSNKRLLRRQEKTDTTNGD...NIRYASMLQDFLNTYHRRGV ALI_PLTG_72
## [2]   632 AECTVDELTEISTIYSEAMTD...SEDPVYPLVKEYSDVVSKHP ALI_PLTG_23
## [3]   474 TRVRKSWTAYTSDEKEIYLSA...FAIVTIGASDGRVRYMNPPS ALI_PLTG_38
## [4]   144 FQQVTMSATTATAITTKLKQF...SMVEHIKTTKRVIDEVQDHV ALI_PLTG_83
## [5]   186 QCSDVHVVFARGSGEAAGLGI...SDGSVTTAAQKAAALVKANA ALI_PLTG_5
```

Alternatively, all the fasta slots could be also retrieved at once with `getFastas` method, inherited from `CBSResult` class:

```
getFastas(step1_sp4)
```

```
## AAStringSetList of length 3
## [["in_fasta"]] ALI_PLTG_98=MSLNYLAHTNMRRARENFLKFLEDEEVEWKYLEACMQRENAALVL...
## [["out_fasta"]] ALI_PLTG_72=MRLKFSILIFGAVLLATTTNADDNSNKRLLRRQEKTDTTNGDEE...
## [["mature_fasta"]] ALI_PLTG_72=DDNSNKRLLRRQEKTDTTNGDEERWNLLSMFKTPNDFTTKI...
```

The remaining slots could be accessed with the designated methods:

```
getSPtibble(step1_sp4)
```

```
## # A tibble: 5 x 9
##   gene_id      Cmax  Cpos  Ymax  Ypos  Smax  Spos Smean Prediction
##   <fct>       <dbl> <int> <dbl> <int> <dbl> <int> <dbl> <chr>
## 1 ALI_PLTG_72 0.739    22 0.794    22 0.948     3 0.851 Signal peptide
## 2 ALI_PLTG_23 0.362    22 0.533    22 0.910    12 0.783 Signal peptide
## 3 ALI_PLTG_38 0.603    22 0.746    20 0.982    10 0.933 Signal peptide
## 4 ALI_PLTG_83 0.179    21 0.338    21 0.843    17 0.640 Signal peptide
## 5 ALI_PLTG_5  0.712    25 0.678    25 0.846    10 0.652 Signal peptide
```

```
getSPversion(step1_sp4)
```

```
## [1] 4
```

Next, imagine we would like to run all the **SignalP** versions on the same input for comparison. We can do this by simply changing value of the `version` argument:

```
step1_sp3 <- signalp(inp,
                    version = 3,
                    organism = 'euk',
                    run_mode = "starter",
                    legacy_method = 'hmm')
## Version used ... signalp3
## Running signalp locally ...
## 2  sequences need to be truncated ...
## Ok for single processing.
## Submitted sequences... 100
## signalp < 4, calling parser for the output ...
## signalp output is imported and filter 'Signal peptide' is applied.
## Import completed!
## Candidate sequences with signal peptides ... 9
step1_sp2 <- signalp(inp,
                    version = 2,
                    organism = 'euk',
                    run_mode = "starter",
                    legacy_method = 'hmm')
## Version used ... signalp2
## Running signalp locally ...
## 2  sequences need to be truncated ...
## Ok for single processing.
## Submitted sequences... 100
## signalp < 4, calling parser for the output ...
## signalp output is imported and filter 'Signal peptide' is applied.
## Import completed!
## Candidate sequences with signal peptides ... 9
step1_sp4 <- signalp(inp,
                    version = 4,
                    organism = 'euk',
                    run_mode = "starter")
## Version used ... signalp4
## Running signalp locally ...
## 2  sequences need to be truncated ...
## Ok for single processing.
## Submitted sequences... 100
## Candidate sequences with signal peptides ... 5
```

In this case application of **SignalP 4.1** version resulted in fewer candidates. Please note, that despite differences in the output format generated by multiple versions of **SignalP**, `signalp()` returns `sp_tibble` in a standard format.

To pipe results from different versions of **SignalP** just switch to the `run_mode = 'piper'` for the second and other downstream steps. In this case `signalp()` will run the analysis using contents of `out_fasta` slot as an input. Say, we want to do the following piping: **signalp2** -> **signalp3** -> **signalp4**.

We will re-use the `step1_sp4` object generated earlier in the `starter` mode for the **signalp4** -> **signalp3** piping operation:

```
step2_sp3 <- signalp(step1_sp2,
                     version = 3,
                     organism = 'euk',
                     run_mode = 'piper',
                     legacy_method = 'hmm')
## Version used ... signalp3
## Running signalp locally ...
## Ok for single processing.
## Submitted sequences... 9
## signalp < 4, calling parser for the output ...
## signalp output is imported and filter 'Signal peptide' is applied.
## Import completed!
## Candidate sequences with signal peptides ... 9
```

Similar with the **signalp3** -> **signalp4** piping:

```
step3_sp4 <- signalp(step2_sp3,
                     version = 4,
                     organism = 'euk',
                     run_mode = "piper")
## Version used ... signalp4
## Running signalp locally ...
## Ok for single processing.
## Submitted sequences... 9
## Candidate sequences with signal peptides ... 5
```

With the input fasta files containing more than 1000 sequences, `signalp()` will automatically switch to parallel mode. It will split the input into smaller chunks and run prediction as a massive parallel process using specified number of CPUs (see `cores` argument). Execution time depends on the number of CPUs available and the input file size. With 48 CPUs it takes ~2 minutes to run `signalp()` on the input fasta file with more than 40’000 sequences.

4.2 Tmhmm()

TMHMM predicts transmembrane α-helices and identifies integral membrane proteins based on HMMs (Krogh *et al.*, 2001; Sonnhammer *et al.*, 1998). It is important to exclude proteins with transmembrane domains located after signal peptide, as they will be retained in the membrane. Since **TMHMM** is often unable to distinguish between N-terminal signal peptides and transmembrane domains, it is recommended to run `tmhmmm()` on the mature sequences obtained after running `signalp()` function.

`tmhmm()` function can handle input objects of `SignalpResult` class with non-empty `mature_fasta` slot.

Here we will use output of the `signalp()` as an input for `tmhmm()`:

```
tm <- tmhmm(step1_sp4, TM = 0)
## Running TMHMM locally ...
## Submitted sequences ... 5
## Candidates with signal peptides and 0 TM domains in mature seq ... 5
```

Attempts to run `tmhmm()` on the `CBSResult` object lacking `mature_fasta` slot will produce an error:

```
tm2 <- tmhmm(inp, TM = 0)
## Error in tmhmm(inp, TM = 0): Input object does not belong to SignalpResult class.
```

`tmhmm()` outputs instances of `TMhmmResult` class, also inheriting from `CBSResult`.

```
class(tm)
```

```
## [1] "TMhmmResult"
## attr(,"package")
## [1] "SecretSanta"
```

```
tm
```

```
## An object of class TMhmmResult
##         in_fasta        out_fasta  in_mature_fasta out_mature_fasta
##                5                5                5                5
## TMHMM tabular output:
## # A tibble: 5 x 6
##   gene_id     length  ExpAA First60 PredHel Topology
##   <chr>        <dbl>  <dbl>   <dbl>   <dbl> <chr>
## 1 ALI_PLTG_5   186   1.29    1.16         0 o
## 2 ALI_PLTG_38  474   0.0900  0.0600       0 o
## 3 ALI_PLTG_23  632   0       0            0 o
## 4 ALI_PLTG_72   97.0 0       0            0 i
## 5 ALI_PLTG_83  144   1.47    0            0 o
```

`TMhmmResult` object contains 5 slots:

- `in_fasta` and `out_fasta` are common slots of all classes inheriting from the `CBSResult` class;
- `in_mature_fasta` - mature sequences used as an input for `tmhmm`
- `out_mature_fasta` - outputted mature sequences not having more transmembrane domains than specified by `TM` threshold.
- `tm_tibble` - parsed **tmhmm** tabular output for candidates not having more transmembrane domains than specified by `TM` threshold;

To get contents of individual slots you could use accessor functions:

```
getTMtibble(tm)
```

```
## # A tibble: 5 x 6
##   gene_id     length  ExpAA First60 PredHel Topology
##   <chr>        <dbl>  <dbl>   <dbl>   <dbl> <chr>
## 1 ALI_PLTG_5   186   1.29    1.16         0 o
## 2 ALI_PLTG_38  474   0.0900  0.0600       0 o
## 3 ALI_PLTG_23  632   0       0            0 o
## 4 ALI_PLTG_72   97.0 0       0            0 i
## 5 ALI_PLTG_83  144   1.47    0            0 o
```

```
getInMatfasta(tm)
```

```
##   A AAStringSet instance of length 5
##     width seq                                          names
## [1]   186 QCSDVHVVFARGSGEAAGLGI...SDGSVTTAAQKAAALVKANA ALI_PLTG_5
```

```
## [2]   474 TRVRKSWTAYTSDEKEIYLSA...FAIVTIGASDGRVRYMNPPS ALI_PLTG_38
## [3]   632 AECTVDELTEISTIYSEAMTD...SEDPVYPLVKEYSDVVSKHP ALI_PLTG_23
## [4]    97 DDNSNKRLLRRQEKTDTTNGD...NIRYASMLQDFLNTYHRRGV ALI_PLTG_72
## [5]   144 FQQVTMSATTATAITTKLKQF...SMVEHIKTTKRVIDEVQDHV ALI_PLTG_83
```

```
getOutMatfasta(tm)
```

```
##   A AAStringSet instance of length 5
##     width seq                                          names
## [1]   186 QCSDVHVVFARGSGEAAGLGI...SDGSVTTAAQKAAALVKANA ALI_PLTG_5
## [2]   474 TRVRKSWTAYTSDEKEIYLSA...FAIVTIGASDGRVRYMNPPS ALI_PLTG_38
## [3]   632 AECTVDELTEISTIYSEAMTD...SEDPVYPLVKEYSDVVSKHP ALI_PLTG_23
## [4]    97 DDNSNKRLLRRQEKTDTTNGD...NIRYASMLQDFLNTYHRRGV ALI_PLTG_72
## [5]   144 FQQVTMSATTATAITTKLKQF...SMVEHIKTTKRVIDEVQDHV ALI_PLTG_83
```

```
getFastas(tm)
```

```
## AAStringSetList of length 4
## [["in_fasta"]] ALI_PLTG_5=MKLSSVCTIIFGLVFIDFNNVVNAQCSDVHVVFARGSGEAAGLGIC...
## [["out_fasta"]] ALI_PLTG_5=MKLSSVCTIIFGLVFIDFNNVVNAQCSDVHVVFARGSGEAAGLGI...
## [["in_mature_fasta"]] ALI_PLTG_5=QCSDVHVVFARGSGEAAGLGICGQPLVSGIAANLPGMSV...
## [["out_mature_fasta"]] ALI_PLTG_5=QCSDVHVVFARGSGEAAGLGICGQPLVSGIAANLPGMS...
```

4.3 Topcons()

SecretSanta provides a way to use an alternative method for prediction of transmembrane domains - TOPCONS, which uses consensus prediction of multiple tools and can more accurately discriminate between signal peptides and transmembrane regions (Tsirigos *et al.*, 2015). Currently there are several options to run TOPCONS: using a web-server, WSDL-API scripts or stand-alone version. Since it takes considerable time to run TOPCONS predictions for a full proteome (from few hours to few days), it makes direct integration of this method in **SecretSanta** wrapper framework not so optimal. Instead we have implemented a parser function `topcons()`, which converts precomputed TOPCONS output into to a compatible `CBSResult` object and can be plugged in in versatile **SecretSanta** pipelines.

Since TOPCONS method itself takes quite a while to run for large protein datasets, `topcons()` parser is restricted to `piper`-like behavior and requires an input from a previous analytic step organised in `CBSResult` object as well as path to directory with generated files. In the example below, we will use precomputed TOPCONS output to convert it to a `TopconsResult` object, again inheriting from `CBSResult`.

```
# first - predict signal peptides
sp <- signalp(inp,
              version = 2,
              organism = 'euk',
              run_mode = "starter",
              legacy_method = 'hmm')
## Version used ... signalp2
## Running signalp locally ...
## 2  sequences need to be truncated ...
## Ok for single processing.
## Submitted sequences... 100
## signalp < 4, calling parser for the output ...
```

```
## signalp output is imported and filter 'Signal peptide' is applied.
## Import completed!
## Candidate sequences with signal peptides ... 9
# extract out_fasta slot with positive candidates and use these sequences to run TOPCONS
prediction on a web-server:
writeXStringSet(getOutfasta(sp), 'sp_out.fasta')
# provide file path/file name with archived results:
p_dir <- system.file("extdata", "rst_SVw4hG.zip", package = "SecretSanta")
# integrate TOPCONS predictions:
tpc <- topcons(input_obj = sp,
               parse_dir = p_dir,
               topcons_mode = "WEB-server",
               TM = 0,
               SP = TRUE)
## Running topcons parser for WEB-server format ...
## Verify SP predictions ... NO
## TM domains allowed ... 0
## Number of result proteins ... 9
tpc
## An object of class TopconsResult
##  in_fasta out_fasta
##         9         9
## Topcons tabular output:
## # A tibble: 9 x 7
##   seq    length    TM SP    source run_time gene_id
##   <fct>   <int> <int> <fct> <fct>     <dbl> <chr>
## 1 seq_4     210     0 True  cached        0 ALI_PLTG_5
## 2 seq_22    653     0 True  cached        0 ALI_PLTG_23
## 3 seq_37    495     0 True  cached        0 ALI_PLTG_38
## 4 seq_58    125     0 True  cached        0 ALI_PLTG_59
## 5 seq_71    118     0 True  cached        0 ALI_PLTG_72
## 6 seq_72    154     0 True  cached        0 ALI_PLTG_73
## 7 seq_80    133     0 True  cached        0 ALI_PLTG_81
## 8 seq_82    164     0 True  cached        0 ALI_PLTG_83
## 9 seq_94   1116     0 True  cached        0 ALI_PLTG_95
```

4.4 Targetp()

TargetP predicts the subcellular localization of secreted eukaryotic proteins based on the presence of signal peptide (SP), chloroplast transit peptides (cTP) or mitochondrial targeting peptides (mTP) in the N-terminus (Emanuelsson *et al.*, 2000). Including **TargetP** in the pipeline can provide additional evidence that a protein with a predicted signal peptide is not targeted to plastids or mitochondria and is indeed extracellular. The `targetp()` function accepts input objects belonging to the `CBSResult` class and uses `out_fasta` slot as a direct input to the **TargetP** method, i.e, predictions are based on the full protein sequences, not their mature versions.

`targetp()` requires specifying `network_type` argument to run correctly. Possible values include:

- *'P'* - for plants;
- *'N'* - for non_plants.

It is possible to run `targetp()` in both *piper* and *starter* modes. Imagine, we want to start our pipeline by running `targetp()` using already created `inp` object as an input:

```r
tp <- targetp(inp,
        network = 'N',  # for non-plant networks
        run_mode = 'starter')
## running targetp locally...
## Ok for single processing
## Number of submitted sequences... 100
## Number of candidate secreted sequences 10
```

Alternatively, we can run `targetp()` on the output of other functions, for example, `signalp()` in the *piper* mode:

```r
tp_pipe <- targetp(step1_sp2,
                   network = 'N',
                   run_mode = 'piper')
## running targetp locally...
## Ok for single processing
## Number of submitted sequences... 9
## Number of candidate secreted sequences 6
```

In both cases `targetp()` will output an object of `TargetpResult` class with the following slots:

```r
class(tp)
```

```
## [1] "TargetpResult"
## attr(,"package")
## [1] "SecretSanta"
```

```r
tp
```

```
## An object of class TargetpResult
##  in_fasta out_fasta
##       100        10
## TargetP tabular output:
## # A tibble: 10 x 7
##    gene_id     length    mTP    sp  other TP_localization    RC
##    <chr>        <int>  <dbl> <dbl>  <dbl> <fct>           <int>
##  1 ALI_PLTG_5     210 0.0270 0.944 0.0960 S                   1
##  2 ALI_PLTG_19    957 0.176  0.359 0.306  S                   5
##  3 ALI_PLTG_23    653 0.0250 0.953 0.0720 S                   1
##  4 ALI_PLTG_38    495 0.123  0.951 0.0120 S                   1
##  5 ALI_PLTG_69    164 0.138  0.527 0.267  S                   4
##  6 ALI_PLTG_72    118 0.0860 0.877 0.0540 S                   2
##  7 ALI_PLTG_73    154 0.0350 0.938 0.108  S                   1
##  8 ALI_PLTG_74    419 0.0240 0.922 0.257  S                   2
##  9 ALI_PLTG_77    347 0.0900 0.475 0.365  S                   5
## 10 ALI_PLTG_81    133 0.134  0.581 0.133  S                   3
```

- `in_fasta` and `out_fasta` are common slots of all classes inheriting from `CBSResult` class;
- `tp_tibble` - parsed **TargetP** tabular output for candidates not targeted to mitochondria or plastids, i.e most likely to be secreted.

To access the `tp_tibble` slot use the `getTPtibble()` method:

```
getTPtibble(tp)
```

```
## # A tibble: 10 x 7
##    gene_id     length    mTP    sp  other TP_localization    RC
##    <chr>        <int>  <dbl> <dbl>  <dbl> <fct>           <int>
##  1 ALI_PLTG_5     210 0.0270 0.944 0.0960 S                   1
##  2 ALI_PLTG_19    957 0.176  0.359 0.306  S                   5
##  3 ALI_PLTG_23    653 0.0250 0.953 0.0720 S                   1
##  4 ALI_PLTG_38    495 0.123  0.951 0.0120 S                   1
##  5 ALI_PLTG_69    164 0.138  0.527 0.267  S                   4
##  6 ALI_PLTG_72    118 0.0860 0.877 0.0540 S                   2
##  7 ALI_PLTG_73    154 0.0350 0.938 0.108  S                   1
##  8 ALI_PLTG_74    419 0.0240 0.922 0.257  S                   2
##  9 ALI_PLTG_77    347 0.0900 0.475 0.365  S                   5
## 10 ALI_PLTG_81    133 0.134  0.581 0.133  S                   3
```

`targetp()` will automatically switch to a parallel mode if the number of input sequences is greater than 1000.

4.5 Wolfpsort()

WoLF PSORT - predicts protein subcellular localization based on the PSORT principle. It converts amino acid sequences into numerical localization features based on sorting signals, amino acid composition and functional motifs. The converted data is then classified based on k-nearest neighbor algorithm (Horton *et al.*, 2006).

`wolfpsort()` requires `organism` argument. Possible values include:

- *'plant'*;
- *'animal'*;
- *'fungi'*.

The `wolfpsort` function accepts objects of `CBSResult` class, and could be run in both `starter` and `piper` modes.

```
wlf <- wolfpsort(step1_sp2, organism = 'fungi', run_mode = 'piper')
## Running WoLF PSORT locally ...
## Number of submitted sequences ... 9
## Candidate sequences with extracellular localisation ... 5
```

`wolfpsort` returns an object with 3 slots:

```
class(wlf)
```

```
## [1] "WolfResult"
## attr(,"package")
## [1] "SecretSanta"
```

```
wlf
```

```
## An object of class WolfResult
##  in_fasta out_fasta
##         9         5
## WoLFPSort tabular output:
## # A tibble: 5 x 2
##   gene_id     localization
##   <fct>       <fct>
## 1 ALI_PLTG_38 extr
## 2 ALI_PLTG_73 extr
## 3 ALI_PLTG_23 extr
## 4 ALI_PLTG_81 extr
## 5 ALI_PLTG_5  extr
```

- `in_fasta` and `out_fasta`;
- `wolf_tibble` - parsed **WoLF PSORT** tabular output for sequences with 'extracellular' predicted to be the most probable subcellular localization.

To access contents of the `wolf_tibble` slot use `getWOlFtibble()` method:

```
getWOLFtibble(wlf)
```

```
## # A tibble: 5 x 2
##   gene_id     localization
##   <fct>       <fct>
## 1 ALI_PLTG_38 extr
## 2 ALI_PLTG_73 extr
## 3 ALI_PLTG_23 extr
## 4 ALI_PLTG_81 extr
## 5 ALI_PLTG_5  extr
```

Since `wolfpsort()` has the same purpose as `targetp()` it might be useful to run these functions side by side to compare the obtained results (see targetp results).

4.6 Check_khdel()

In addition to having signal peptides, some proteins can have an ER-retention signal in the C-terminal domain that prevents protein from being secreted outside the cell. There are at least 2 known ER-retention motifs (*KDEL* and *HDEL*) (Munro and Pelham, 1987). Currently, several definitions of ER-retention signal exist, `check_khdel()` function allows the user to specify one of 3 possible definitions with `pattern` argument:

- `pattern = 'prosite'` - checking for ER-retention motifs defined as "[KRHQSA][DENQ]EL$>";
- `pattern = 'elm'` - checking for ER-retention motifs defined as "[KRHQSAP][DENQT]EL$;
- `pattern = 'strict'` - strict matching for N-terminal *KDEL* or *HDEL* motifs;

The `check_khdel()` uses pattern matching, according to the specified definition to scan amino acid sequences and remove those with an ER retention signal in the C-terminus. To run it, simply pass a `CBSResult` object as an input.
`check_khdel()` is restricted to piper-like behavior, because it makes the most sense to check for ER-retention signals in proteins with already predicted signal peptides. The function does not rely on external dependencies, so providing path container is not required.

```
er_result <- check_khdel(step1_sp2, pattern = 'prosite')
## Checking for terminal ER retention signals ... prosite pattern.
```

```
## Submitted sequences ... 9
## Sequences with terminal ER retention signals detected ... 0
## Candidate without terminal ER retention signals detected ... 9
```

This will return an object with 3 slots:

```
er_result
```

```
## An object of class ErResult
##       in_fasta      out_fasta retained_fasta
##              9              9              0
```

- `in_fasta` and `out_fasta`;
- `retained_fasta` - all the sequences with C-terminal '(K/H)DEL' motifs, in case you would like to have a look at them.

4.7 M_slicer()

This is an experimental option. The `m_slicer()` function takes the input amino acid sequences and generates all possible subsequences starting with methionine based on the assumption that translation start sites might be mis-predicted in the original set of proteins, which in turn would result in signal peptides also being mis-predicted. The output of this step can be used as an input for secretome prediction pipelines to rescue secreted proteins with mis-predicted start sites. (Making a list and checking it twice…just like Santa does). Sequence ids for the newly generated slices are built by concatenating original sequence ids, 'slice' string and position of the methionine sliced from. For example: `ALI_PLTG_3_slice_M86` means that `ALI_PLTG_3` sequence was sliced starting from the methionine in the position 86.

`m_slicer()` has 2 running modes:

- `slice` - to simply slice input fasta regardless of it's origin;

```
slice <- m_slicer(aa,  # a set of amino acid seqeunces
                run_mode = 'slice',
                min_len = 100 # minimal length of the outputed slices
)
```

- `rescue` - having output from any other up-stream function it extracts proteins not predicted to be secreted on the initial run and generate slices for them.

```
rescued <- m_slicer(step1_sp2,  # signalp2 output
                run_mode = 'rescue',
                min_len = 100)
```

Results of both run modes could be used as an input for other `SecretSanta` predictors. Say, we want to run `signalp()` on the rescued proteins. Note, that `m_slicer()` outputs `AAStringSet` objects, so in order to pass it to `signalp()` we first need to create `CBSResult` object with `in_fasta = rescued`.

```
r_inp <- CBSResult(in_fasta = rescued)
sp_rescue <- signalp(r_inp,
                    version = 2,
                    organism = 'euk',
                    run_mode = 'starter',
```

```
                  truncate = TRUE,
                  legacy_method = 'hmm')
## Version used ... signalp2
## Running signalp locally ...
## Ok for single processing.
## Fasta size exceedes maximal total residue limit, seqs >  430  residues will be
truncated.
## 192  sequences need to be truncated ...
## Submitted sequences... 527
## signalp < 4, calling parser for the output ...
## signalp output is imported and filter 'Signal peptide' is applied.
## Import completed!
## Candidate sequences with signal peptides ... 24
```

The slicing procedure returned 24 additional potentially secreted candidates.

4.8 Ask_uniprot()

`ask_uniprot()` is a convenience function, allowing to retrieve information on subcellular localisation of proteins from UniProtKB in order to compare results obtained within a **SecretSanta** pipeline with available information on subcellular localisation. This function is mostly relevant for well-annotated and curated genomes. `ask_uniprot()` currently requires a simple list of UniprotKB ids and returns information about subcellular localisation, key words as well as relevant GO terms organised in a tibble:

```
id_list <- c('P39864', 'D0N4E2', 'Q5BUB4', 'D0N381', 'B1NNT7', 'D0NP26')
res <- ask_uniprot(id_list)
```

```
## Fetching location from UniprotKB ...
```

```
res
```

```
## # A tibble: 6 x 4
##   UniprotID Subcellular.Location                         Key.Words   GO.CC
##   <chr>     <chr>                                        <chr>       <chr>
## 1 P39864    not present                                  Disulfide … ""
## 2 D0N4E2    not present                                  ATP-bindin… GO:0…
## 3 Q5BUB4    Nucleus {ECO:0000305}.                       DNA-bindin… GO:0…
## 4 D0N381    Cytoplasm {ECO:0000255|HAMAP-Rule:MF_03115}. 2Fe-2S; Ap… GO:0…
## 5 B1NNT7    Secreted {ECO:0000305|PubMed:19694952}.      Repeat; Se… GO:0…
## 6 D0NP26    not present                                  Complete p… GO:0…
```

5. Build pipelines

We are now ready to build a pipeline using all of our available methods. Say, we would like to have a relatively short pipeline (**Figure 3**) with the following analytic steps:

- use **SignalP4** to predict signal peptides and cleavage sites;
- run **TMHMM** on the output, to ensure that predicted proteins with signal peptides do not contain TM domains, i.e won't be stuck in the membrane;
- run **TargetP** on the output, to make sure that a set of selected proteins is not targeted to plastids or mitochondria;

- collect the **TargetP** output and scan for ER-retention signals - to ensure that proteins won't be stuck in the ER.

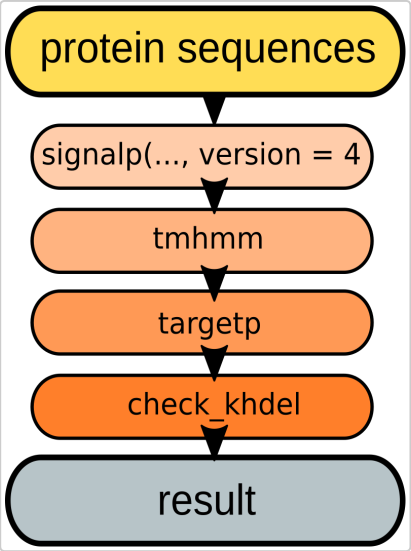

Figure 3: Minimal pipeline for secretome prediction

Now we will run this pipeline using `SecretSanta` functions, starting with `aa` - an `AAStringSet` object containing 100 amino acid sequences.

Step 1: predict signal peptides

```
input <- CBSResult(in_fasta = aa)

input
## An object of class CBSResult
##  in_fasta out_fasta
##       100         0
step1_sp4 <- signalp(input,
                    version = 4,
                    organism = 'euk',
                    run_mode = 'starter',
                    truncate = TRUE
                    )
## Version used ... signalp4
## Running signalp locally ...
## 2  sequences need to be truncated ...
## Ok for single processing.
## Submitted sequences... 100
## Candidate sequences with signal peptides ... 5

getSPtibble(step1_sp4)
## # A tibble: 5 x 9
##   gene_id      Cmax  Cpos  Ymax  Ypos  Smax  Spos Smean Prediction
##   <fct>       <dbl> <int> <dbl> <int> <dbl> <int> <dbl> <chr>
## 1 ALI_PLTG_83 0.179    21 0.338    21 0.843    17 0.640 Signal peptide
## 2 ALI_PLTG_72 0.739    22 0.794    22 0.948     3 0.851 Signal peptide
## 3 ALI_PLTG_23 0.362    22 0.533    22 0.910    12 0.783 Signal peptide
## 4 ALI_PLTG_38 0.603    22 0.746    20 0.982    10 0.933 Signal peptide
## 5 ALI_PLTG_5  0.712    25 0.678    25 0.846    10 0.652 Signal peptide
```

```
getOutfasta(step1_sp4)
##   A AAStringSet instance of length 5
##     width seq                                               names
## [1]   164 MVSTSRVFALLLLPSPSARIF...SMVEHIKTTKRVIDEVQDHV ALI_PLTG_83
## [2]   118 MRLKFSILIFGAVLLATTTNA...NIRYASMLQDFLNTYHRRGV ALI_PLTG_72
## [3]   653 MKIVALVTFCIATLDSSIVFA...SEDPVYPLVKEYSDVVSKHP ALI_PLTG_23
## [4]   495 MVVRVLLVVLALLAVGVQSKA...FAIVTIGASDGRVRYMNPPS ALI_PLTG_38
## [5]   210 MKLSSVCTIIFGLVFIDFNNV...SDGSVTTAAQKAAALVKANA ALI_PLTG_5

getMatfasta(step1_sp4)
##   A AAStringSet instance of length 5
##     width seq                                               names
## [1]   144 FQQVTMSATTATAITTKLKQF...SMVEHIKTTKRVIDEVQDHV ALI_PLTG_83
## [2]    97 DDNSNKRLLRRQEKTDTTNGD...NIRYASMLQDFLNTYHRRGV ALI_PLTG_72
## [3]   632 AECTVDELTEISTIYSEAMTD...SEDPVYPLVKEYSDVVSKHP ALI_PLTG_23
## [4]   474 TRVRKSWTAYTSDEKEIYLSA...FAIVTIGASDGRVRYMNPPS ALI_PLTG_38
## [5]   186 QCSDVHVVFARGSGEAAGLGI...SDGSVTTAAQKAAALVKANA ALI_PLTG_5
```

Result: 5 candidate proteins with signal peptides.

Step 2: check for presence of TM domains in mature peptides:

```
# we allow 0 TM domains in mature peptides
step2_tm <- tmhmm(step1_sp4, TM = 0)
## Running TMHMM locally ...
## Submitted sequences ... 5
## Candidates with signal peptides and 0 TM domains in mature seq ... 5

getTMtibble(step2_tm)
## # A tibble: 5 x 6
##   gene_id     length  ExpAA First60 PredHel Topology
##   <chr>        <dbl>  <dbl>   <dbl>   <dbl> <chr>
## 1 ALI_PLTG_83  144   1.47    0             0 o
## 2 ALI_PLTG_72   97.0 0       0             0 i
## 3 ALI_PLTG_23  632   0       0             0 o
## 4 ALI_PLTG_38  474   0.0900  0.0600        0 o
## 5 ALI_PLTG_5   186   1.29    1.16          0 o

getOutfasta(step2_tm)
##   A AAStringSet instance of length 5
##     width seq                                               names
## [1]   164 MVSTSRVFALLLLPSPSARIF...SMVEHIKTTKRVIDEVQDHV ALI_PLTG_83
## [2]   118 MRLKFSILIFGAVLLATTTNA...NIRYASMLQDFLNTYHRRGV ALI_PLTG_72
## [3]   653 MKIVALVTFCIATLDSSIVFA...SEDPVYPLVKEYSDVVSKHP ALI_PLTG_23
## [4]   495 MVVRVLLVVLALLAVGVQSKA...FAIVTIGASDGRVRYMNPPS ALI_PLTG_38
## [5]   210 MKLSSVCTIIFGLVFIDFNNV...SDGSVTTAAQKAAALVKANA ALI_PLTG_5

getOutMatfasta(step2_tm)
##   A AAStringSet instance of length 5
```

```
##     width seq                                         names
## [1]   144 FQQVTMSATTATAITTKLKQF...SMVEHIKTTKRVIDEVQDHV ALI_PLTG_83
## [2]    97 DDNSNKRLLRRQEKTDTTNGD...NIRYASMLQDFLNTYHRRGV ALI_PLTG_72
## [3]   632 AECTVDELTEISTIYSEAMTD...SEDPVYPLVKEYSDVVSKHP ALI_PLTG_23
## [4]   474 TRVRKSWTAYTSDEKEIYLSA...FAIVTIGASDGRVRYMNPPS ALI_PLTG_38
## [5]   186 QCSDVHVVFARGSGEAAGLGI...SDGSVTTAAQKAAALVKANA ALI_PLTG_5
```

> **Result**: 5 candidate proteins with signal peptides; 0 TM domains in mature sequences.

Step 3: predict sub-cellular localization

Here we are using `targetp`, we could have also used `wolfpsort`.

```
step3_tp <- targetp(step2_tm, network = 'N',run_mode = 'piper')
## running targetp locally...
## Ok for single processing
## Number of submitted sequences... 5
## Number of candidate secreted sequences 4

getTPtibble(step3_tp)
## # A tibble: 4 x 7
##   gene_id     length    mTP    sp  other TP_localization    RC
##   <chr>        <int>  <dbl> <dbl>  <dbl> <fct>           <int>
## 1 ALI_PLTG_72    118 0.0860 0.877 0.0540 S                   2
## 2 ALI_PLTG_23    653 0.0250 0.953 0.0720 S                   1
## 3 ALI_PLTG_38    495 0.123  0.951 0.0120 S                   1
## 4 ALI_PLTG_5     210 0.0270 0.944 0.0960 S                   1

getInfasta(step3_tp)
##   A AAStringSet instance of length 5
##     width seq                                         names
## [1]   164 MVSTSRVFALLLLPSPSARIF...SMVEHIKTTKRVIDEVQDHV ALI_PLTG_83
## [2]   118 MRLKFSILIFGAVLLATTTNA...NIRYASMLQDFLNTYHRRGV ALI_PLTG_72
## [3]   653 MKIVALVTFCIATLDSSIVFA...SEDPVYPLVKEYSDVVSKHP ALI_PLTG_23
## [4]   495 MVVRVLLVVLALLAVGVQSKA...FAIVTIGASDGRVRYMNPPS ALI_PLTG_38
## [5]   210 MKLSSVCTIIFGLVFIDFNNV...SDGSVTTAAQKAAALVKANA ALI_PLTG_5

getOutfasta(step3_tp)
##   A AAStringSet instance of length 4
##     width seq                                         names
## [1]   118 MRLKFSILIFGAVLLATTTNA...NIRYASMLQDFLNTYHRRGV ALI_PLTG_72
## [2]   653 MKIVALVTFCIATLDSSIVFA...SEDPVYPLVKEYSDVVSKHP ALI_PLTG_23
## [3]   495 MVVRVLLVVLALLAVGVQSKA...FAIVTIGASDGRVRYMNPPS ALI_PLTG_38
## [4]   210 MKLSSVCTIIFGLVFIDFNNV...SDGSVTTAAQKAAALVKANA ALI_PLTG_5
```

This step allowed us to filter 1 sequence potentially targeted to mitochondria.

> **Result**: 4 candidate proteins with signal peptides; 0 TM domains in mature sequences; not targeted to plastids or mitochondria.

Step 4: check for ER-retention signals

```
step4_er <- check_khdel(step3_tp, 'elm')
## Checking for terminal ER retention signals ... elm pattern.
## Submitted sequences ... 4
## Sequences with terminal ER retention signals detected ... 0
## Candidate without terminal ER retention signals detected ... 4
getOutfasta(step4_er)
##   A AAStringSet instance of length 4
##     width seq                                          names
## [1]   118 MRLKFSILIFGAVLLATTTNA...NIRYASMLQDFLNTYHRRGV ALI_PLTG_72
## [2]   653 MKIVALVTFCIATLDSSIVFA...SEDPVYPLVKEYSDVVSKHP ALI_PLTG_23
## [3]   495 MVVRVLLVVLALLAVGVQSKA...FAIVTIGASDGRVRYMNPPS ALI_PLTG_38
## [4]   210 MKLSSVCTIIFGLVFIDFNNV...SDGSVTTAAQKAAALVKANA ALI_PLTG_5
```

> **Final Result**: 4 candidate proteins with signal peptides; 0 TM domains in mature sequences; not targeted to plastids or mitochondria; not retained in ER.

This is an example of a fairly simple pipeline, but you can create more complex ones. A more stringent pipeline might involve multi-step filtration on signal peptide prediction as well as additional predictions for 'rescued' (**Figure 4**) sequences.

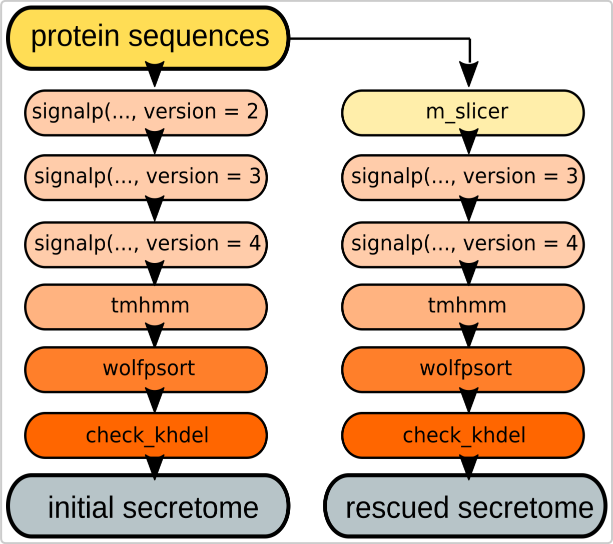

> **Figure 4**: Example of a stringent pipeline.

Step 5: save the result secretome

```
writeXStringSet(getOutfasta(step4_er), 'secretome_out.fasta')
```

Compact pipelienes:

Now, once we have examined all the individual analytic steps, we can directly combine them in a single command by using `%>%` operator:

```
pipeline <- signalp(input, version = 4, organism = 'euk', run_mode = 'starter') %>%
    tmhmm(TM = 0) %>%
    targetp(network = 'N', run_mode = 'piper') %>%
    check_khdel(pattern = 'prosite')
## Version used ... signalp4
## Running signalp locally ...
## 2  sequences need to be truncated ...
## Ok for single processing.
## Submitted sequences... 100
## Candidate sequences with signal peptides ... 5
## Running TMHMM locally ...
## Submitted sequences ... 5
## Candidates with signal peptides and 0 TM domains in mature seq ... 5
## running targetp locally...
## Ok for single processing
## Number of submitted sequences... 5
## Number of candidate secreted sequences 4
## Checking for terminal ER retention signals ... prosite pattern.
## Submitted sequences ... 4
## Sequences with terminal ER retention signals detected ... 0
## Candidate without terminal ER retention signals detected ... 4
```

Since `pp` belongs to the `CBSResult` class, we can again extract the output fasta field and save it in a separate file:

```
writeXStringSet(getOutfasta(pipeline), 'secretome_out.fasta')
```

Use case: predict secretome for a model species and compare with annotations available in UniProtKB

In this example we will download proteome for *Phytophthora infestans* from the UniprotKB, construct a pipeline for secretome prediction and compare results with known annotations. We will use **TOPCONS** for prediction of transmembrane domains instead of **TMHMM**, so to save time we will import already precomputed results and integrate them in the pipeline. To retrieve proteome, we will use the most recent version of **biomartr** package (Hajk-Georg and Jerzy, 2017).

```
devtools::install_github("HajkD/biomartr")
library(biomartr)

#download P.infestans proteome from UniProtKB

Pinfestans.proteome.uniprot <- getProteome(db = "uniprot",
                                           organism = "Phytophthora infestans",
                                           path = "_uniprot_downloads/proteomes")

# Create CBSResult class object and start a pipeline:
pinf <- CBSResult(in_fasta = readAAStringSet(Pinfestans.proteome.uniprot))

# TOPCONS predictions for P.infestans:
top_dir <- system.file("extdata", "rst_7gaVh2.zip", package = "SecretSanta")

# Predict signal peptides, check additional targeting signals and ER-motifs, integrate
```

```
TOPCONS predictions of TM domains
Pinfestans.secretome <- signalp(pinf,
                      version = 4,
                      organism = 'euk',
                      sensitive = TRUE,
                      run_mode = 'starter',
                      cores = 3) %>%
              targetp(network = 'N', run_mode = 'piper') %>%
              check_khdel(pattern = 'prosite') %>%
              topcons(parse_dir = top_dir, topcons_mode = "WEB-server",
                      TM = 0, SP = TRUE)

# Now, we can check how many of predicted proteins have already known extracellular
localisation, according to UniProtKB:
unip_res <- ask_uniprot(names(getOutfasta(Pinfestans.secretome)))
table(unip_res$Subcellular.Location)
```

6. Running pipelines on HPCs

To demonstrate how **SecretSanta** pipelines could be applied for large-scale analysis of proteomes, we will download all available fungal proteomes using **biomartr** package (Hajk-Georg and Jerzy, 2017) and apply the same standard secretome prediction pipeline. To speed-up the process, we will use 20 cores available on our local HPC. Please note, retrieval of proteomes might take a while.

```
library(biomartr)
meta.retrieval(kingdom = "fungi", db = "refseq", type = 'proteome',
               path = "fungi_refseq_proteomes")

proteomes <- list.files("fungi_refseq_proteomes/",
                        full.names = TRUE, pattern ="*.fasta")

hpc_pipeline <- function(proteome) {
    input <- CBSResult(in_fasta = readAAStringSet(proteome))
    pipeline <- signalp(input, version = 4, organism = 'euk',
                        run_mode = 'starter', cores = 20) %>%
        tmhmm(TM = 0) %>%
        targetp(network = 'N', run_mode = 'piper', cores = 20) %>%
        check_khdel(pattern = 'prosite')
    base_proteome <- stringr::str_split(proteome, '/') %>% tail(n = 1)
    writeXStringSet(getOutfasta(pipeline),
                    paste(base_proteome, 'secretome.fasta',
                          sep = '_'))

}

sapply(proteomes, hpc_pipeline)
```

Session

```
sessionInfo()
```

```
## R version 3.4.0 (2017-04-21)
## Platform: x86_64-pc-linux-gnu (64-bit)
## Running under: Ubuntu 16.04.2 LTS
##
## Matrix products: default
## BLAS: /usr/lib/openblas-base/libblas.so.3
## LAPACK: /usr/lib/libopenblasp-r0.2.18.so
##
## locale:
##  [1] LC_CTYPE=en_GB.UTF-8       LC_NUMERIC=C
##  [3] LC_TIME=en_GB.UTF-8        LC_COLLATE=en_GB.UTF-8
##  [5] LC_MONETARY=en_GB.UTF-8    LC_MESSAGES=en_GB.UTF-8
##  [7] LC_PAPER=en_GB.UTF-8       LC_NAME=C
##  [9] LC_ADDRESS=C               LC_TELEPHONE=C
## [11] LC_MEASUREMENT=en_GB.UTF-8 LC_IDENTIFICATION=C
##
## attached base packages:
## [1] stats4    parallel  stats     graphics  grDevices utils     datasets
## [8] methods   base
##
## other attached packages:
## [1] bindrcpp_0.2        SecretSanta_0.99.0  stringr_1.2.0
## [4] dplyr_0.7.4         Biostrings_2.44.2   XVector_0.16.0
## [7] IRanges_2.10.3      S4Vectors_0.14.7    BiocGenerics_0.22.1
##
## loaded via a namespace (and not attached):
##  [1] Rcpp_0.12.14     rstudioapi_0.7   knitr_1.17       bindr_0.1
##  [5] magrittr_1.5     zlibbioc_1.22.0  R6_2.2.2         rlang_0.1.6.9003
##  [9] highr_0.6        httr_1.3.1       tools_3.4.0      utf8_1.1.3
## [13] cli_1.0.0        htmltools_0.3.6  yaml_2.1.15      rprojroot_1.2
## [17] digest_0.6.12    assertthat_0.2.0 tibble_1.4.2     crayon_1.3.4
## [21] curl_3.1         glue_1.1.1       evaluate_0.10.1  rmarkdown_1.8.3
## [25] stringi_1.1.5    pillar_1.1.0     compiler_3.4.0   backports_1.1.0
## [29] pkgconfig_2.0.1
```

References

Bendtsen,J. *et al.* (2004) Improved prediction of signal peptides: SignalP 3.0. *J. Mol. Biol.*, **340**, 783–795.

Emanuelsson,O. *et al.* (2000) Predicting subcellular localization of proteins based on their N-terminal amino acid sequence. *J. Mol. Biol.*, **300**, 1005–1016.

Evangelisti,E. *et al.* (2017) Time-resolved dual transcriptomics reveal early induced Nicotiana benthamiana root genes and conserved infection-promoting Phytophthora palmivora effectors. *BMC Biology*, **15:39**.

Hajk-Georg,D. and Jerzy,P. (2017) Biomartr: Genomic data retrieval with R. *Bioinformatics*.

Horton,P. *et al.* (2006) Protein subcellular localization prediction with WoLF PSORT. *Asian Pacific Bioinformatics Conference, APCB2006*, 39–48.

Krogh,A. *et al.* (2001) Predicting transmembrane protein topology with a hidden Markov model: Application to complete genomes. *J. Mol. Biol.*, **305**, 567–580.

Munro,S. and Pelham,H. (1987) A C-terminal signal prevents secretion of luminal ER proteins. *Cell*, **48**, 899–907.

Nielsen,H. and Krogh,A. (1998) Prediction of signal peptides and signal anchors by a hidden Markov model. *Proceedings of the Sixth International Conference on Intelligent Systems for Molecular Biology*

(ISMB 6), 122–130.

Nielsen,H. *et al.* (1997) Identification of prokaryotic and eukaryotic signal peptides and prediction of their cleavage sites. *Protein Engineering*, **10**, 1–6.

Petersen,T. *et al.* (2011) SignalP 4.0: Discriminating signal peptides from transmembrane regions. *Nature Methods*, **8**, 785–786.

Sonnhammer,E. *et al.* (1998) A hidden Markov model for predicting transmembrane helices in protein sequences. *In J. Glasgow et al., eds.: Proc. Sixth Int. Conf. on Intelligent Systems for Molecular Biology*, 175–182.

Sperschneider,J. *et al.* (2015) Evaluation of secretion prediction highlights differing approaches needed for Oomycete and Fungal effectors. *Front Plant Sci*, **6**, 1168.

Tsirigos,K. *et al.* (2015) The TOPCONS web server for combined membrane protein topology and signal peptide prediction. *Nucleic Acids Research*, **43**, w401–w407.
